# Claims-based Analysis of Zoster Vaccine Effectiveness against Incident Herpes Zoster: The VENUS Study

**DOI:** 10.31662/jmaj.2025-0027

**Published:** 2025-09-26

**Authors:** Fumiko Murata, Megumi Maeda, Haruhisa Fukuda

**Affiliations:** 1 Department of Health Care Administration and Management, Graduate School of Medical Sciences, Kyushu University, Fukuoka, Japan

**Keywords:** vaccine effectiveness, herpes zoster, recombinant zoster vaccine, varicella vaccine, real-world data

## Introduction

Herpes zoster (HZ) is a neurocutaneous disease caused by the reactivation of latent varicella zoster virus in sensory ganglia ^(1)^. In Japan, a 21-year study in Miyazaki reported an HZ incidence of 6.07 per 1,000 person-years in 2017 ^(2)^, and another study found an incidence of 10.9 per 1,000 person-years among adults aged 50 years and older ^(3)^. HZ incidence increases with age, with lifetime risk of 50% by age 85 years ^(4)^. As the incidence of HZ is expected to rise in Japan’s aging population, its prevention has become an important public health goal.

Numerous countries recommend the recombinant zoster vaccine (RZV) Shingrix (GSK) to prevent the development of HZ in adults aged ≥50 years ^(5)^. A randomized, placebo-controlled, phase 3 trial reported that the efficacy of RZV against incident HZ was 97.2% in adults aged ≥50 years ^(6)^. In 2016, Japan’s Ministry of Health, Labour and Welfare approved the use of Biken (BIKEN Co., Ltd.), a freeze-dried varicella vaccine live (VVL), as a HZ vaccine ^(7)^. A study in Japan reported that VVL reduced HZ incidence by 27.8% in adults aged ≥50 years ^(8)^. To the best of our knowledge, no other studies have assessed the effectiveness of VVL in Japan.

Despite HZ vaccines effects demonstrated in clinical trials, little is known about their real-world effectiveness in Japan. This study aimed to assess the effectiveness of HZ vaccines against incident HZ in two Japanese municipalities.

## Methods

### Study design and settings

This retrospective matched cohort study used data from the Longevity Improvement & Fair Evidence (LIFE) Study ^(9)^. The LIFE Study regularly receives insurance claims data from over 30 participating municipalities. These municipalities submit anonymized data from residents enrolled in Japan’s National Health Insurance and Latter-Stage Older Persons Health Care System. National Health Insurance is a public medical insurance program that covers the self-employed, unemployed, irregularly employed, primary industry workers, and retirees aged <75 years. The Latter-Stage Older Persons Health Care System is a public medical insurance program that covers persons aged ≥75 years and persons aged 65-74 years with specific debilitating diseases. These insurance programs generate claims data that include information on each enrollee’s diagnoses, procedures, and use of health resources during insurance-covered health care encounters. The Vaccine Effectiveness, Networking, and Universal Safety (VENUS) Study was created as a sub-project of the LIFE Study, with the aim of supporting research on vaccine effectiveness and safety in Japan ^(10)^. The VENUS Study collects and merges vaccination records (vaccines, vaccination dates, doses, manufacturers, and lot numbers) with the LIFE Study’s claims data. In Japan, the HZ vaccine is voluntary. Some municipalities provide subsidies and maintain vaccination records. This study focused on two municipalities (designated City A and City B) participating in the LIFE Study that independently subsidize the HZ vaccine.

### Study population

Because both target municipalities partially subsidize HZ vaccinations for adults aged ≥50 years, we focused on residents aged ≥50 years during fiscal year 2022 (i.e., April 2022 to March 2023). Using vaccination records, we identified their HZ vaccination statuses during this period.

In City A, 99.4% of HZ vaccine recipients had received RZV, and only 0.6% had received VVL; therefore, we focused only on RZV recipients in that municipality. In City B, 76.7% of HZ vaccine recipients had received VVL, and only 23.4% had received RZV; therefore, we focused only on VVL recipients in that municipality. Each recipient’s date of RZV or VVL vaccination between April 2022 and March 2023 was set as his/her cohort entry date (CED). The date of the first RZV dose was defined as the CED; however, participants who received only one dose were excluded from the analysis.

Next, each vaccine recipient was randomly matched to an unvaccinated resident to create a matched cohort, based on municipality, sex, and age at the CED. The unvaccinated residents were assigned the same CED as their vaccinated counterparts, ensuring that the comparison between the two groups was made at equivalent baseline time points.

We excluded vaccinated and unvaccinated residents who were not enrolled in either the National Health Insurance or Latter-Stage Older Persons Health Care System in the 6 months before their CED.

### Study exposure and outcome

The study exposure was HZ vaccination status (vaccinated vs unvaccinated), and the outcome was incident HZ after the CED. Observations were censored at the study’s end (March 2023) or the last claims record. Unvaccinated residents were censored if they received the HZ vaccine. HZ was identified using the following International Classification of Diseases, 10th Revision codes: B020, B021, B022, B023, B027, B028, and B029 ^(11), (12)^.

### Statistical analysis

First, we described the participants’ characteristics using mean and standard deviation for age and percentages for sex. These characteristics were ascertained from the claims data. Next, we compared HZ incidence between vaccinated and unvaccinated individuals using McNemar’s test (vaccinated/unvaccinated and HZ/no HZ), with matched pairs determined based on municipality, sex, and CED. The mid-p-value was calculated to account for small sample sizes and provide a more accurate estimate ^(13)^.

The statistical analyses were conducted using Stata version 17.0 (Stata Corp, College Station, TX), and significance was set at p < 0.05. The study was approved by the Kyushu University Institutional Review Board for Clinical Research (Approval No. 22114-02).

## Results

The participant characteristics are summarized in Table 1. In City A, 294 RZV-vaccinated participants were matched with 294 unvaccinated participants. Among these, 67.7% were women, and the mean age was 74.9 years (standard deviation: 7.5 years). In City B, 197 participants vaccinated with VVL were matched with 197 unvaccinated participants. Among these, 72.6% were women, and the mean age was 74.7 years (standard deviation: 6.7 years).

**Table 1.** Characteristics of Participants.

	Unvaccinated	RZV-vaccinated	Unvaccinated	VVL-vaccinated
n	294	294	197	197
Mean follow-up duration (months) [SD]	5.3 [1.6]	5.5 [1.6]	5.1 [2.3]	5.3 [2.3]
Women, n (%)	199 (67.7)	199 (67.7)	143 (72.6)	143 (72.6)
Mean age (years) [SD]	74.9 [7.5]	74.9 [7.5]	74.7 [6.7]	74.7 [6.7]

RZV: recombinant zoster vaccine; SD: standard deviation; VVL: varicella vaccine live.

Table 2 shows the results of McNemar’s test for RZV and VVL. There were no HZ cases in RZV-vaccinated participants and 5 HZ cases (1.7%) in unvaccinated participants. RZV-vaccinated participants had a notable trend toward a lower incidence of HZ than the unvaccinated participants (χ^2^ = 5.00, mid-p = 0.06). Next, there were 2 HZ cases (1.0%) in VVL-vaccinated participants and 4 HZ cases (2.0%) in unvaccinated participants. VVL-vaccinated participants showed no significant difference in HZ incidence compared with unvaccinated individuals (χ^2^ = 1.00, mid-p = 0.22).

**Table 2.** Associations Between Zoster Vaccination and Incident HZ.

	Unvaccinated	RZV-vaccinated	Mid-p	Unvaccinated	VVL-vaccinated	Mid-p
Incident HZ, n (%)	5 (1.7)	0 (0.0)	0.06	4 (2.0)	2 (1.0)	0.22
Mean duration until HZ occurrence (months) [SD]	3.4 [2.9]	NA		4.3 [1.7]	6 [2.8]	

p values were calculated using McNemar’s test. HZ: herpes zoster; NA: not applicable; RZV: recombinant zoster vaccine; SD: standard deviation; VVL: varicella vaccine live.

## Discussion

Using a database combining claims data and vaccination records, this retrospective matched cohort study examined the effectiveness of HZ vaccination in reducing HZ incidence in two Japanese municipalities. The results showed that although no statistically significant differences were observed between RZV and VVL recipients and unvaccinated individuals, there was a notable trend toward lower HZ incidence among vaccine recipients (mid-p = 0.06), suggesting a potential protective effect.

A claims-based cohort study in the United States calculated RZV effectiveness against incident HZ to be 85.5% in adults aged ≥50 years ^(14)^. Similarly, a prospective cohort study in the United States found that two RZV doses had an effectiveness of 79% in preventing HZ in adults aged ≥50 years for 1 year after the second dose ^(11)^. The only Japanese study on VVL effectiveness reported that this vaccine reduced incident HZ by 27.8% in adults aged ≥50 years over a median follow-up period of 3.36 years ^(8)^. A randomized, placebo-controlled, double-blind clinical trial of Zostavax (Merck & Co.)―using the same Oka-strain varicella zoster virus as VVL―found that this vaccine reduced HZ incidence by 51.3% in adults aged ≥60 years over a median follow-up period of 3.12 years ^(15)^. Our study found that RZV was associated with a notably lower incidence of HZ, although the difference did not reach conventional statistical significance, whereas VVL showed no statistically significant difference compared with unvaccinated individuals. To the best of our knowledge, no studies in Japan have examined the effectiveness of RZV using real-world data. Little is known about VVL’s effectiveness in Japan. This study provides key insight into the real-world effectiveness of RZV and VVL against incident HZ. However, given the small sample size observed in this study, careful interpretation of the results is warranted.

This study has several limitations. First, the study was conducted using data from only two municipalities, limiting generalizability. Second, vaccinated participants were identified using vaccination records provided by municipalities. However, HZ vaccination is voluntary in Japan, and these records would not include those immunized without subsidy. Therefore, it is possible that unvaccinated groups may have included some vaccinated individuals, potentially underestimating vaccine effectiveness. In addition, individuals who cannot receive the vaccine due to certain medical conditions are not identifiable in these records, further complicating the assessment of vaccine effectiveness. Third, our study did not account for underlying conditions, which could influence vaccination patterns. Fourth, the study used insurance claims data, which lack important clinical information, such as HZ severity. Fifth, although we have some information on comorbidities, because of the small number of outcome events, we faced constraints in adequately adjusting for confounding variables. Furthermore, we do not have data on socioeconomic status and health-seeking behavior. This limitation may impact the validity of our findings and underscores the need for cautious interpretation of the results. Finally, the relatively short follow-up period of approximately half a year may have limited our ability to detect later-onset cases or potential waning of vaccine effectiveness.

Despite these limitations, our study presents evidence on the real-world effectiveness of HZ vaccinations against incident HZ in Japan. HZ vaccines are not routinely administered in Japan. However, an expert advisory panel of the Ministry of Health, Labor and Welfare convened in June 2024 and agreed that it was appropriate to include these vaccines in Japan’s routine immunization program ^(16)^. HZ incidence is expected to rise with Japan’s aging population. Along with providing subsidies, the introduction of routine immunization programs is expected to increase the number of vaccinated individuals, which will facilitate more comprehensive and robust evaluations of vaccine effectiveness in the future.

### Conclusion

With zero infections in the vaccinated group, the RZV vaccine was suggested to be effective in preventing infection, although statistical significance could not be demonstrated because of the small sample size.

## Article Information

### Acknowledgments

The authors are grateful to the staff of the two participating municipalities for their cooperation in this study.

### Author Contributions

Fumiko Murata, Megumi Maeda, and Haruhisa Fukuda designed the study and collected the data. Fumiko Murata performed the analysis, and all authors interpreted the results. Fumiko Murata drafted the original manuscript. All authors reviewed and edited the manuscript. The study was supervised by Haruhisa Fukuda. All authors read the manuscript and approved its submission for publication.

### Conflicts of Interest

None

### IRB Approval Code and Name of the Institution

Approval number 22114-02 and the Kyushu University Institutional Review Board for Clinical Research.

### Data Sharing

Data cannot be shared for privacy or ethical reasons.
